# America First, Africa Last? Health data deals and the new scramble for pathogens

**DOI:** 10.1371/journal.pgph.0005974

**Published:** 2026-02-23

**Authors:** Sharifah Sekalala, Shajoe J. Lake, Allan Maleche, Timothy Wafula

**Affiliations:** 1 Centre for Global Health Law, Warwick School of Law, Coventry, United Kingdom; 2 KELIN, Nairobi, Kenya; PLOS: Public Library of Science, UNITED STATES OF AMERICA

On 4 December 2025, Kenya became the first country to sign a five-year bilateral global health agreement (BGHA) called a Health Cooperation Framework with the US, worth about US$1.6–2.5 billion and presented as a bold move to strengthen HIV, TB, malaria, and pandemic preparedness, including surveillance and workforce reforms [1]. Soon after, Rwanda and Uganda signed similar BGHAs worth US$228 million and US$2.3 billion, respetcively [2]. These agreements are explicitly branded as part of an “America First” global health strategy, under which dozens of similar bilateral deals are expected across Africa [1].

The official explanation is that the US needs near real-time access to Kenyan health data, including outbreak surveillance, to protect global health security and guide its investments [3]. The BGHAs give US agencies access to digital health systems and outbreak databases, and authority to audit a sample of facilities, in exchange for large-scale funding that is framed as helping these countries achieve “health sovereignty” [4]. A separate US template for BGHAs, reported in November, asked partner countries to share biological specimens and genetic sequences of pathogens with epidemic potential within days of detection, with specimen-sharing commitments lasting up to 25 years but without guaranteed reciprocal access to any vaccines or treatments later developed. Here, health data and pathogen access are the price of re-entering US funding circuits after earlier aid cuts.

Kenyan officials claim that only aggregated data will be shared and that Kenyan law, including the Data Protection Act and Digital Health Act, formally prevails over the agreement [5]. However, in practice, when core systems, hosting, and analytics are controlled through foreign-designed architectures and contracts, local regulators face a steep uphill battle to supervise what happens once health data migrates [6].

Kenya’s High Court has intervened, issuing conservatory orders that temporarily suspend implementation of the agreement “insofar as it provides for or facilitates the transfer, sharing or dissemination” of medical, epidemiological, or sensitive personal health data, following petitions by the Consumers Federation of Kenya and Senator Okiya Omtatah [5]. This may seem like a dispute over privacy, but it is an early constitutional test of whether African courts will treat health data as a strategic asset whose governance cannot be outsourced through executive diplomacy.

BGHAs also need to be read against the wider political economy of pathogen sharing. In 2025, WHO member states adopted a Pandemic Agreement, which includes a Pathogen Access and Benefit Sharing (PABS) system, now being negotiated as an annex [7]. PABS aims to secure rapid sharing of pathogen samples and sequence data, in exchange for more equitable access to vaccines, diagnostics, and other countermeasures. The US, having withdrawn from WHO and the Pandemic Agreement process, is instead advancing its own bilateral model, tying restoration of health aid to bespoke obligations to supply pathogen data.

Yet this is the tip of the iceberg. These BGHAs are early nodes in an alternative PABS framework, built through bilateral bargains that resemble “TRIPS-plus” trade deals. Instead of a multilateral system where African regional blocs can negotiate binding benefit-sharing rules collectively, individual countries are pushed to accept terms under severe fiscal pressure and aid uncertainty. BGHAs risk normalising a practice where data and pathogen access are governed by fragmented bilateral treaties, while benefit sharing remains aspirational and uneven.

For African nations, the danger is not only legal fragmentation but the consolidation of long-standing extractive relations through research and data infrastructures. Decades of global health scholarship have documented how North–South research collaborations, are structured by unequal funding relations that concentrate agenda-setting power, epistemic authority, and control over outputs in the Global North [8]. Historical accounts link present-day health research in Africa to these dynamics, documenting patterns of unequal authorship, asymmetrical benefit-sharing, and research designs shaped more by external scientific priorities than local health needs [9].

Recent reporting on a US-funded hepatitis B vaccine study in Guinea-Bissau renders these relations even more visible. The trial, funded by the US CDC and implemented through the Bandim Health Project, plans to enrol approximately 14,000 newborns, randomising infants to receive the hepatitis B vaccine either at birth or at six weeks, despite established WHO recommendations and high local disease prevalence and ethical objections from leading scientific experts [10].

Commentators in Kenya have already warned that health data and genomic information are becoming the new frontiers of a scramble for African resources [11]. Pathogen samples, genomic databases, and longitudinal electronic health records are all inputs for AI tools, pharmaceutical pipelines, and security analytics that will generate economic and strategic value far beyond Africa. Yet the communities whose bodies and clinics feed these systems are unlikely to hold intellectual property, shape research agendas, or reliably access the resulting products.

Race is crucial to this arrangement. Countries such as Keyna, Rwanda, and Uganda are asked to trade health data and pathogens for funding under the language of “partnership”, while the principal gains from expanded surveillance capacities, patent portfolios, and AI tools continue to accrue to the US and its allied markets. The insistence that these frameworks end dependency by moving toward country ownership obscures the fact that ownership of the most valuable asset—data—is being reconfigured, not returned. The result is digital colonialism; African lives are counted and monitored to manage risk elsewhere, under the banner of partnership [12].

The immediate harms may feel abstract to many patients. A clinic visit still looks like a conversation with a nurse rather than a contribution to a training dataset. But over time, the stakes become tangible. Insurers using predictive analytics to classify people as uninsurable, employers screening workers through health-risk scoring, or security agencies targeting “risky” border populations using syndromic data. None of this is inevitable, but the legal and technical infrastructure being assembled now will shape what is possible in the future and who gets to decide.

The High Court’s stay in Kenya shows that domestic law and public mobilisation can slow the advance of extractive health data regimes. But national safeguards alone are insufficient. A coordinated African position among governments and institutions like Africa CDC is urgently needed. One that demands BGHAs be transparent, subject to parliamentary approval, aligned with emerging PABS rules, and tied to enforceable commitments on benefit-sharing, technology transfer, and public control of digital infrastructure. No existing model fully satisfies these requirements, but instructive partial precedents exist. H3Africa foregrounded African ownership in genomic research governance, GISAID pioneered conditional and attribution-based data-sharing, and the NHS Federated Data Platform offered (and later compromised) a model of public oversight [13–15]. These cases reveal both the institutional possibilities and political risks of treating health data as a collective asset. Civil society should use this moment to insist that health cooperation be negotiated on similar terms.

If these agreements are allowed to roll out across the continent without such safeguards, they will have done more than restore some of the funding stripped by recent US cuts. They will have quietly rewritten the terms on which African bodies, clinics, and laboratories are enrolled into the next generation of global health security. The fight in Kenya is therefore not only about one country’s privacy law but an early confrontation over whether the future of pandemic preparedness will be shaped by multilateralism, or by a series of bilateral contracts where those who hold capital set the rules and those who hold patients and pathogens carry the risk.
